# SECONDGRAM: Self-conditioned diffusion with gradient manipulation for longitudinal MRI imputation

**DOI:** 10.1016/j.patter.2025.101212

**Published:** 2025-03-31

**Authors:** Brandon Theodorou, Anant Dadu, Mike Nalls, Faraz Faghri, Jimeng Sun

**Affiliations:** 1 Department of Computer Science, University of Illinois at Urbana-Champaign, Urbana, IL, USA; 2 Center for Alzheimer’s and Related Dementias, National Institutes of Health, Bethesda, MD, USA; 3 Laboratory of Neurogenetics, National Institute on Aging, National Institutes of Health, Bethesda, MD, USA; 4 Data Tecnica International, LLC, Washington, DC, USA

**Keywords:** machine learning in healthcare, generative modeling, diffusion models, imputation, augmentation, longitudinal MRI, neurodegenerative diseases

## Abstract

While individual MRI snapshots provide valuable insights, the longitudinal progression in repeated MRIs often holds more significant diagnostic and prognostic value. However, a scarcity of longitudinal datasets, comprising paired initial and follow-up scans, hinders the application of machine learning for crucial sequential tasks. We address this gap by proposing self-conditioned diffusion with gradient manipulation (SECONDGRAM) to generate absent follow-up imaging features, enabling predictions of MRI developments over time and enriching limited datasets through imputation. SECONDGRAM builds on neural diffusion models and introduces two key contributions: self-conditioned learning to leverage much larger, unlinked datasets and gradient manipulation to combat instability and overfitting in a low-data setting. We evaluate SECONDGRAM on the UK Biobank dataset and show that it not only models MRI patterns better than existing baselines but also enhances training datasets to achieve better downstream results over naive approaches.

## Highlights

• SECONDGRAM accurately imputes missing longitudinal MRI data
• Self-conditioning effectively uses abundant unpaired MRI data
• Gradient manipulation robustly prevents model overfitting
• SECONDGRAM-imputed data boost downstream diagnostic accuracy

## The bigger picture

Longitudinal MRI is a type of medical imaging study where MRI scans are taken several times over a period of time to track changes. These studies play a pivotal role in understanding neurodegenerative diseases like Alzheimer’s disease and Parkinson’s disease, yet the limited availability of paired longitudinal imaging datasets significantly restricts advanced machine learning applications. Our research introduces SECONDGRAM, a robust framework utilizing neural diffusion models enhanced with self-conditioned learning and gradient manipulation techniques. These models are computer algorithms that simulate how information changes. SECONDGRAM addresses data scarcity by generating realistic follow-up MRI imaging features, thereby enriching limited datasets. This methodological advancement not only improves the accuracy and realism of imputed imaging features but also significantly boosts the performance of predictive models in critical downstream tasks. The implications extend beyond healthcare, presenting a versatile solution for data augmentation in numerous fields struggling with longitudinal data constraints, thus enhancing decision-making processes in precision medicine.

## Teaser

Longitudinal MRI datasets essential for neurodegenerative disease research are scarce, limiting advanced machine learning approaches. The authors address this challenge with a neural diffusion-based framework, SECONDGRAM, that can generate realistic follow-up MRI features. Leveraging self-conditioned learning and robust gradient manipulation, SECONDGRAM significantly enhances data quality and improves predictive performance for medical diagnoses, offering broad applicability in precision medicine and beyond.

## Introduction

Magnetic resonance imaging (MRI) is an indispensable tool in modern medicine, providing pivotal insights into various medical conditions.^1^ These include finding early signs of Parkinson’s disease,^2^^,^^3^ monitoring tumors,^4^ and evaluating soft tissue injuries.^5^ However, beyond mere individual snapshots, the longitudinal progression depicted in repeated MRI results, capturing changes over time, often holds more significant diagnostic and prognostic value.^6^^,^^7^ This temporal trajectory is crucial for anticipating disorders like Alzheimer’s disease,^8^^,^^9^ tracking the progression of conditions like multiple sclerosis,^10^ and assessing the effectiveness of treatments.^11^

The surge of machine learning (ML) applications in healthcare for tasks such as drug discovery,^12^ health monitoring,^13^ and clinical predictive modeling^14^^,^^15^ presents a compelling opportunity to leverage these techniques for analyzing MRI images. Therefore, a number of approaches have been developed to harness recent advances toward tasks such as MRI reconstruction,^16^ MRI segmentation,^17^^,^^18^ and disease prediction and classification using MRI images.^19^^,^^20^ Corresponding efforts have also been made to automate the process of MRI feature extraction^19^^,^^21^^,^^22^ to improve such analytic and ML approaches by allowing them to operate over structured features and measurements rather than raw images.

However, while there exist multiple large MRI datasets^23^^,^^24^^,^^25^ that can be used to train such models for tasks centering around a single MRI, there is a dearth of longitudinal MRI datasets comprising paired initial and follow-up scans. This data limitation has become a bottleneck in harnessing ML for crucial sequential tasks.

To address this gap, our research seeks to develop a model capable of generating absent follow-up imaging features. This will then let us both predict MRI developments over time and perform imputation on limited datasets to enrich them and allow for the training of better models on longitudinal tasks using MRI pairs. To do this, we will need to address the key challenges of this generative modeling task:

(1) conditioning on previous MRI results that are anatomically similar but slightly altered,
(2) handling an extremely limited number of paired images to discern the patterns of progression, and
(3) making use of a wealth of isolated MRI results that do not have any paired, follow-up images.

To that end, we propose self-conditioned diffusion with gradient manipulation (SECONDGRAM), which uses the current state-of-the-art approach to image generation, neural diffusion models, with a pair of key contributions aimed at our specific problem setting:

(1) self-conditioned learning to leverage the available large, nonlongitudinal datasets for training by conditioning MRI generations on their possibly noised selves and
(2) gradient manipulation to fight instability and overfitting in the face of limited data using gradient norm clipping and heuristic gradient skipping.

We evaluate SECONDGRAM using the popular UK Biobank dataset, one of the largest long-term biomedical databases in the world. We show not only that SECONDGRAM is able to model both overall and temporal MRI patterns better than existing baselines but also that this follow-up imaging imputation approach is able to achieve better downstream modeling results through enhanced training datasets than the naive approaches of either training on a limited amount of temporal data or training on a large amount of non-sequential data. Specifically, SECONDGRAM achieves a 20% improvement in mean Pearson correlation between the imputed and real follow-up image features in a held-out test set over the nearest baseline and then offers up to a 10% improvement in downstream stroke classification area under the receiving operating characteristic curve (AUROC) for models trained on its enriched datasets versus naive approaches.

## Results

### Problem formulation

#### Definition 1 (imaging features)

We let $i\in R^{n_{i}}$ represent the features derived from an MRI scan using a pipeline such as in Tustison et al.^21^ These features are generally defined to capture the content of an image, including statistics regarding the volumes, areas, and thicknesses of various subcortical brain structures and other minutiae, in a structured format. Each variable in i is normalized to lie within a defined range (we set this range to be [−1,1]). For longitudinal studies, we define $i_{1}$ to be the imaging features from the first visit and $i_{2}$ to be the features from a second, follow-up visit. While our architecture and experiments focus on derived MRI features measuring various different brain regions and structures, we note that our framework is compatible with any modality of MRI features or even the original image itself.

#### Definition 2 (patient labels)

We then denote the additional patient-specific labels as $l\in R_{n_{l}}$. These include demographic variables (in our experiments, we use gender, age at recruitment, and Townsend deprivation index) and medical outcomes (including a variety of disease prognoses and mortality information). These labels serve dual roles: conditioning imaging data for generation and acting as potential predictors for downstream models, especially in the case of the disease and outcome variables.

#### Task 1 (follow-up imaging generation)

The task is then, given $i_{1}$ and l, to generate $i_{2}$ so that it closely mirrors the true progression between MRI results. This is achieved by learning and sampling from $P\left(i_{2}|i_{1},l\right)$.

### Experimental design

We evaluate our method and compare it to several baselines comprising other logical conditional generative architectures in a series of experiments on the UK Biobank dataset.^26^ Specifically, we aim to answer the following questions.

(1) Can SECONDGRAM closely approximate real follow-up data?
(2) Does SECONDGRAM capture the distributions of MRI imaging features as a whole?
(3) Does SECONDGRAM’s generative abilities allow it to enrich training datasets by imputing follow-up imaging for patients without them?
(4) Does SECONDGRAM’s synthetically generated follow-up imaging demonstrate known patterns in a real-world case study?

We note that we perform all evaluations operating over the imaging features that comprise our dataset as opposed to over any raw imaging results.

### Datasets, baselines, and experimental setup

#### Dataset

We use the real-world UK Biobank dataset, one of the largest long-term biomedical databases in the world and popularly used in a variety of research settings, in our evaluation.^26^ The dataset contains 41,706 patients, each of whom participated in a longitudinal study over multiple years, including an introductory survey and subsequent T1-weighted MRI imaging. 4,997 of the patients then had follow-up MRI imaging performed, on average, 2.67 ± 1.17 years after the first MRI.

Although the time gap between scans is an important variable—and including it in the model could improve the quality of generated follow-up images, especially when comparing to ground truth—it is often unavailable when follow-up scans are missing. As a result, incorporating time gaps into the model and task definitions would limit our ability to impute missing follow-up scans in datasets without consistent temporal information. To maintain flexibility, we omit time gaps from our modeling and instead allow the generative model to probabilistically simulate a plausible follow-up MRI without being tied to a specific time interval.

For each volumetric T1-weighted MRI, we leverage the preprocessed measurements released in UK Biobank’s tabular dataset to extract 769 anatomical measurement features, such as the volume, thickness, and area of different brain structures and regions. This pipeline allows the measurement of features from specific and small subcortical regions, such as the globus pallidus and substantia nigra, in order to paint a complete and detailed picture of the MRI image content in structured vector form. These features are then min-max normalized to the range [−1,1] and imputed with scikit-learn’s^27^ iterative multivariate imputer in the rare case of missingness before serving as our final imaging feature vector i. These feature vectors then serve as our imaging dataset, acting as both input and output format for our compared methods and serving as the centerpiece of all evaluations. Finally, we extract a series of 14 demographic and disease variables, such as age, gender, Townsend deprivation index, parkinsonism, and stroke history from the initial survey, and add a mortality variable signaling death by the end of the study for use as our auxiliary patient labels in terms of both model input and downstream evaluation.

We randomly split the patients with follow-up imaging into training and test datasets with an 80-20 ratio, adding all patients without follow-up imaging to the training set to be used for self-conditioned learning. We then further split off 10% of this combined training set into a validation set. The final dataset sizes are presented in Table 1. Using these datasets, we train our models within the PyTorch framework^28^ for 1,000 epochs (with a patience cutoff of 50 epochs) at a 0.001 learning rate and using the Adam optimizer. We save the model that performs best on the validation set, as determined by its generations being closest to the true follow-up imaging there, and evaluate it using the test set.

**Table 1:** Final dataset sizes

Dataset	Longitudinal	Non-longitudinal
Training	3,597	33,038
Validation	400	3,671
Testing	1,000	0

#### Baselines

We consider the following baseline models. Note that each method compared here is conditioned on the same information (the first set of imaging features $i_{1}$ and auxiliary patient labels l) while aiming to generate the follow-up imaging features $i_{2}$.

(1) The general adversarial network (GAN) is a conditional Wasserstein GAN^29^ trained on the limited follow-up imaging data available.
(2) Vanilla diffusion is a standard conditional diffusion model trained on the limited follow-up imaging data available.
(3) Pretrained diffusion is a diffusion model that is first pretrained on the wealth of unconditional MRI imaging features before being fine-tuned on the limited conditional, follow-up imaging data available.

#### Ablation models

To demonstrate the value of SECONDGRAM’s contributions, we add two ablation baselines, which are trained on the combined dataset but without any gradient manipulation and with that gradient manipulation but on the limited, conditioned dataset. We call these models “w/o grad” and “w/o self,” respectively.

#### Experimental setup

To conduct our experiments, we train 100 different models with random seeds 1 through 100 for each of our compared methods. For each model, we then generate follow-up MRI results for each patient in each of our datasets in order to perform a variety of analyses, including comparing the similarity of imputed follow-up results for patients with real follow-up imaging to enrich training and validation datasets by imputing follow-up imaging features where they are lacking. We report 95% confidence intervals for each experimental value for each metric, calculated using the standard errors of those values across the 100 experimental runs. We present the best values in bold for each metric, and we bold any other values whose means fall into that best value’s confidence interval. We perform all experimentation using NVIDIA P100 GPUs on the National Institutes of Health’s Biowulf cluster (see https://hpc.nih.gov for reference). All source code for our experiments can be found at https://github.com/btheodorou99/SECONDGRAM/.

### Approximating follow-up imaging

The primary objective of our model, SECONDGRAM, is to closely approximate real follow-up data. To assess this, we examine the similarity between imputed follow-up imaging features and the true features for patients in the test set. Table 2 presents a comparative analysis among different methods.

**Table 2:** Similarity between real and imputed imaging features

	Pearson correlation (↑)	Euclidean distance (↓)	Cosine distance (↓)
GAN	0.429 ± 0.021	6.82 ± 0.43	0.309 ± 0.015
Vanilla diffusion	0.500 ± 0.013	8.81 ± 0.29	0.380 ± 0.014
Pretrained diffusion	0.493 ± 0.014	8.42 ± 0.10	0.373 ± 0.010
w/o grad	0.537 ± 0.016	7.64 ± 0.21	0.333 ± 0.015
w/o self	0.494 ± 0.006	8.60 ± 0.08	0.387 ± 0.014
SECONDGRAM	0.601 ± 0.018	6.95 ± 0.33	0.282 ± 0.019

There, we show the mean Pearson correlation, Euclidean distance, and cosine distance between real and generated follow-up imaging feature vectors, each calculated per patient and averaged across the test set. These three metrics collectively offer a nuanced and comprehensive evaluation of feature similarities. Euclidean distance measures absolute scale similarity between features, effectively measuring the “straight-line” distance between points in the feature space. However, it can be overly simplistic in high-dimensional spaces, as that scale outweighs any more intricate variable patterns. Pearson correlation then assesses univariate correlation, measuring how individual variables in two feature sets correlate and move together. It offers a measure that is globally scale invariant but locally shape dependent so that individual outlier values can impede strong performance. Finally, cosine distance measures the difference in the “direction” of two feature vectors, capturing complex covariate patterns but ignoring scale altogether.

Using these metrics, we see that SECONDGRAM performs extremely well, outpacing all of the baseline approaches. While the GAN baseline is extremely effective at generating realistically scaled variables, as shown by its excellent performance in terms of the Euclidean distance, it fails to produce nuanced and coherently multivariate feature sets. This results in a clear gap between it and SECONDGRAM in the cosine distance metric and a much weaker mean Pearson correlation than any of the diffusion-based methods. The pretrained and vanilla diffusion baselines offer limited performance across the board, while SECONDGRAM outperforms each compared method on each metric. Specifically, it matches the GAN baseline in Euclidean distance and slightly exceeds it (by 9%) in terms of cosine distance before improving the Pearson correlation by 20% over the next closest non-ablation baseline. The performance of the two ablation baselines then showcases the clear impact of our two contributions, as removing either greatly limits performance to only narrowly surpass the vanilla diffusion baseline. Thus, we see that SECONDGRAM successfully approximates real follow-up MRI data.

### Capturing overall data distributions

Moving beyond individual imaging feature similarities, the ability of a generative model to reproduce the distribution of a dataset is essential, especially in a medical context, where deviations from the real data distributions can make the data obviously fake. So, we continue to measure not just the similarity between imputed and real follow-up data but also the distribution of each of the 769 variables comprising the imaging result features across the entire imputed datasets and training dataset they were designed to mimic.

In Table 3, we see that the GAN baseline is again able to mimic the simple, top-level patterns of variable scale extremely effectively, as evident from the high Pearson correlation between its synthetic means and the true training means of each variable. Nonetheless, SECONDGRAM is able to approach the GAN baseline’s performance there, and we can see the impact of its contributions from its clear outpacing of both the ablation and other diffusion baselines.

**Table 3:** Real vs. synthetic distribution similarity

	Fréchet distance (↓)	Pearson mean correlation (↑)
GAN	45.873 ± 10.15	0.976 ± 0.006
Vanilla diffusion	46.824 ± 6.35	0.825 ± 0.016
Pretrained diffusion	36.217 ± 1.06	0.853 ± 0.008
w/o grad	31.446 ± 2.68	0.915 ± 0.012
w/o self	45.766 ± 3.25	0.821 ± 0.013
SECONDGRAM	26.088 ± 5.62	0.940 ± 0.011

**Figure 1:**
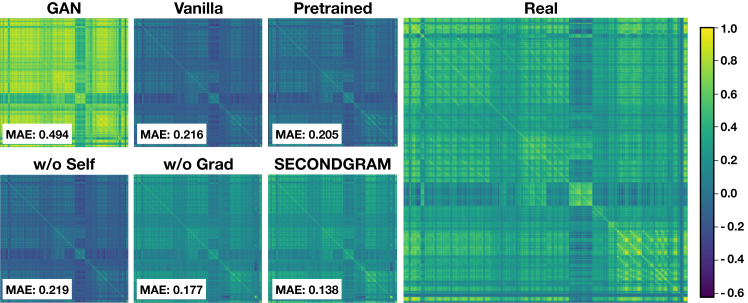
Covariate correlation matrices for real and imputed follow-up imaging datasets Shown are covariate correlation matrices in the real and imputed follow-up imaging training datasets. SECONDGRAM best captures the true covariate distribution patterns over 33% closer than any of the non-ablation baselines. Furthermore, we see how poorly the GAN baseline does at capturing these more intricate patterns, as it has each feature far too correlated to one another despite the individual feature means being very close to the true training means.

Moreover, when assessing more intricate patterns, like the Fréchet distance (replacing the inception model embeddings in the typical Fréchet inception distance score calculation with our MRI feature vectors) in that same table and the covariate distribution as shown in Figure 1, the GAN baseline performs much more poorly, leaving it well behind each of the diffusion models due to overfitting and overcorrelation. SECONDGRAM’s contributions instead enable it to outperform each compared method, striking a balance between capturing general univariate distributions and the nuanced multivariate patterns between the correlating features. It achieves a 28% lower Fréchet distance and 33% closer covariate correlation similarity to the real data than any non-ablation baseline, with significant improvement over each ablation as well. This establishes the capability of SECONDGRAM in capturing the overall MRI distribution, ensuring that generated imaging features aren’t just superficially similar but also share underlying statistical properties with real MRI results.

### Enriching downstream training datasets

Our third evaluation then builds on the first two and explores the true utility of the imputed data. So, we examine the downstream effects of enhancing training datasets when real follow-up images are sparse.

To perform this evaluation, we use tasks defined by our auxiliary patient labels l, which are derived from non-MRI patient records. Specifically, we explore the tasks of parkinsonism and stroke detection classification labeled by the presence of such a diagnosis at any point in a patient’s history. We present these results in Table 4.

**Table 4:** Dataset enrichment utility

	Parkinsonism AUROC (↑)	Stroke AUROC (↑)
All first	0.927 ± 0.011	0.535 ± 0.008
Limited paired	0.911 ± 0.003	0.664 ± 0.004
GAN	0.916 ± 0.016	0.652 ± 0.013
Vanilla diffusion	0.946 ± 0.008	0.710 ± 0.008
Pretrained diffusion	0.947 ± 0.010	0.705 ± 0.008
w/o grad	0.951 ± 0.008	0.732 ± 0.007
w/o self	0.944 ± 0.010	0.709 ± 0.007
SECONDGRAM	0.957 ± 0.007	0.733 ± 0.006

Each experiment is performed using a three-layer neural network model with a hidden dimension of 256, a batch size of 128, a learning rate of 0.001 using the Adam optimizer, and an epoch-level patience hyperparameter of 5.

We first train models via the naive approaches of using all patients but only the initial imaging result features and using only the subset of patients with paired results. In many tasks, especially in rare disease tasks with class imbalances such as the parkinsonism detection, even these models can perform well. However, they are wasteful by definition, either throwing away the vast majority of data points or preventing any longitudinal information from being used.

So, we then repeat the two tasks with datasets that have been enriched with each of our compared methods. There, we keep each patient in the training dataset, and we impute the follow-up imaging features if they do not have it present. Note that this imputation is being conditioned on the task labels via their presence in l, so the models are able to ensure that the appropriate patterns are being modeled and included. We note that, while this conditioning “leaks” the labels to the generation models, this is constrained to the training dataset for the purpose of more accurate augmentation. The downstream model is then trained on this augmented dataset before being deployed to a new test set where the labels are not leaked and the generative model no longer plays any role. Conceptually, these models are attempting to learn $P\left(i_{2}|i_{1},l\right)$ so that downstream models can better learn and sample from $P\left(l|i_{1},i_{2}\right)$ in the face of limited paired $\left(i_{1},i_{2}\right)$ data.

Each method besides the GAN baseline offers at least some level of utility in undergoing this process, showcasing the inherent value of the follow-up imaging generation task as a whole, as it can be used to enhance the performance over naive downstream models. SECONDGRAM then offers the most improvement on each classification task, as measured by downstream prediction AUROC, and it improves over the best naive approach by up to 10%.

This shows that SECONDGRAM not only accurately imputes follow-up imaging features but also adds substantial value to training datasets. The enriched datasets, when used in downstream tasks, result in better predictive performance on a variety of tasks. So, we have shown that SECONDGRAM outperforms the baselines in closely approximating real follow-up data, capturing overall data distributions, and enhancing downstream training datasets, establishing its potential as a powerful tool in the medical imaging domain.

### Parkinsonism case study

Finally, we conclude our evaluation of SECONDGRAM with a case study exploring its ability to learn and replicate known, disease-based patterns. Specifically, we explore whether it captures the correlation between Parkinson’s disease and the decay of a small region of the brain called the substantia nigra and specifically its pars compacta (SNC) subregion.^30^^,^^31^^,^^32^ To do this, we extract all patients in each of our datasets with a real follow-up visit. We then generate synthetic follow-up imaging result features using each of our SECONDGRAM models and examine the 6 variables corresponding to different volume calculations of the SNC subregion, rescaled back to their original value ranges before being averaged into a single volume measure. We analyze both the true and generated changes in this volume and compare the average results across patients with and without a history of parkinsonism based on their auxiliary patient labels, l. We present the findings in Table 5.

**Table 5:** SNC volume changes based on the presence of parkinsonism

	Parkinsonism mean volume change	No parkinsonism mean volume change
Real	−2.36 mm^3^	−1.76 mm^3^
SECONDGRAM	−4.54 mm^3^	−3.49 mm^3^

There, we see that SECONDGRAM is able to learn and recreate this pattern despite the fact that such patients are extremely rare (just 107 cases throughout our entire dataset). While both populations show decay in the SNC volume over time, that decay is noticeably greater in patients with a history of parkinsonism for both the real and SECONDGRAM-generated imaging features. Furthermore, while SECONDGRAM’s degeneration in general is slightly larger across both groups, it nearly perfectly matches the real data in having patients with parkinsonism decay by 30% more, on average, than patients without. The ratio in the true data is a nearly identical 34% more for patients with parkinsonism.

**Figure 2:**
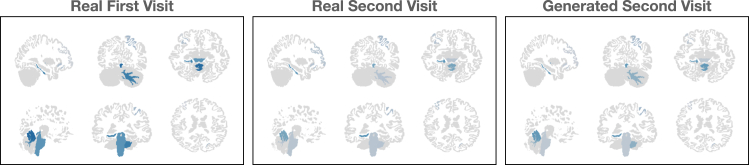
Parkinsonism case study visualizations Shown are visualizations of real and SECONDGRAM-generated MRI results for a patient with a history of parkinsonism. We extract a set of brain regions that are associated with parkinsonism and map the imaging features representing the volume of those regions onto templated brain images to demonstrate that SECONDGRAM is realistically capturing the decay of those regions.

We furthermore show these results visually in Figure 2 by mapping the measurements back onto an MRI template with 9 parkinsonism-associated brain regions (chosen using Shapley values derived from a Parkinson’s disease prediction model) lit up in blue, with their intensity corresponding to the magnitude of the measurement. We do this for one patient with parkinsonism, depicting the original, real follow-up, and SECONDGRAM-generated follow-up MRIs. There, we can see that the patient with parkinsonism is showing clear deterioration of a number of brain regions, a pattern that is captured by the SECONDGRAM model. So, we show that SECONDGRAM is able to capture real, known patterns of brain and MRI progressions over time. This allows its generations to both look more realistic and also provide utility to clinicians and ML models trained to recognize such patterns.

## Discussion

In this study, we present SECONDGRAM, a method for conditionally generating follow-up imaging features from initial imaging results and patient details. Addressing the scarcity of longitudinal imaging data, SECONDGRAM employs a conditional diffusion framework boosted by two main contributions: leveraging the abundance of unconditioned images through a self-conditioning strategy and adopting a gradient manipulation process for enhanced robustness. Our experiments on the UK Biobank dataset show that SECONDGRAM outperforms other baselines in terms of effective imputation, general imaging feature generation, and downstream utility via imputative dataset enhancement. It is able to closely approximate the measurements of the real follow-up imaging and, in doing so, enrich training datasets on a variety of downstream tasks, allowing models to use all available patients without preventing longitudinal modeling. This task and methodology then open up a number of exciting possibilities for not just simulating realistic follow-up MRI features and augmenting downstream training datasets through imputation but even more. While we were constrained to individual MRI pairs by our dataset, this approach and its simulation could easily be extended to handle a flexible number of MRIs, which may yield improved generation and also open the door to additional applications. So, we feel that SECONDGRAM both achieves exciting results showing the feasibility and utility of longitudinal MRI feature generation and also establishes a robust foundation for future explorations in the realm of medical imaging, providing a versatile tool and approach that offers great promise in repairing and augmenting datasets to complete medical records and assist downstream tasks.

## Methods

### Background and related works

#### Image generation

The field of image generation has witnessed significant recent growth and advancement, largely beginning with the use of GANs.^29^^,^^33^^,^^34^ GANs have gained widespread attention for their ability to generate realistic images by training a generator to create data indistinguishable from real samples while a discriminator tries to differentiate between real and generated data. Despite their success, GANs have run into challenges such as mode collapse and training instability.

A more recent and promising approach to image generation is diffusion models. Diffusion models repeatedly refine an image from random noise to the desired target. They have shown the ability to effectively generate high-quality images and capture complex data distributions through this iterative process. Several papers laid down the theoretical and empirical potential of such score-based models,^35^^,^^36^^,^^37^^,^^38^ but the recent swell in popularity has largely accompanied the increasing scale of such models and their training data to achieve incredibly realistic and creative results.^39^^,^^40^ Many of these even generate conditioned images, often on a textual description^39^ or partial observation^41^^,^^42^ that can be taken as at least conceptually similar to the image-based conditioning required for our imputation task. However, it is still an open question how to approximate or even approach such performance when we have more limited training data.

Finally, we note that GAN^43^ and even diffusion^44^^,^^45^^,^^46^ models have been growing in their application within the field of ML in healthcare. This includes generating both MRI data^44^^,^^45^ and other modalities, such as positron emission tomography scans^47^ and X-rays.^48^ However, here, too, we run into the critical challenge of a need for more work exploring follow-up imaging and the challenge of limited paired data.

#### Gradient manipulation in deep learning

Modern deep learning techniques, such as those for image generation, rely on gradient computations. These gradients, derived from an objective function with respect to the model’s parameters, are used to optimize the loss function through iterative updates.

However, gradient-related challenges can hamper the training process.^49^^,^^50^ Notably, vanishing^51^ and exploding^52^ gradients can slow down or destabilize training, respectively. Various solutions span from architectural to data-driven approaches. This paper explores direct gradient manipulation. Among these techniques, gradient clipping caps gradients at a predefined threshold. This has become a key approach to counteract explosive gradients^53^ and has shown its efficacy in training,^54^ especially when confronted with noisy labels.^55^ Beyond clipping, several advanced gradient adjustment strategies are introduced to tackle challenges in multi-objective^56^ and multi-class^57^ scenarios, among others.

#### Medical image prediction and augmentation

While the task of longitudinal image generation is relatively underexplored, with the only other work in the medical domain we could identify being GAN-based image imputation on paired infant MRI scans,^58^ there are a number of similar tasks and ideas that are related.

Numerous works have made more standard longitudinal predictions based on a series of medical images. For example, Jin et al.^59^ predict cancer treatment responses from longitudinal X-ray images. Similarly, Han et al.^60^ predict survival outcomes based on a series of computed tomography scans, and Cascarano et al.^61^ survey other similar applications. While these works do not involve image generation, they do use longitudinal images as data, showcasing the importance of such datasets.

To the other end of our task, there have also been a number of works focusing on enhancing medical image datasets, even if they do not focus on longitudinal images as we do. These efforts focus largely on augmentation to effectively expand datasets. These vary from standard augmentation^62^^,^^63^ to generative model-based augmentation via GANs^64^ and other models.^65^

### SECONDGRAM

Our proposed SECONDGRAM method begins with a canonical conditional diffusion setup augmented by a pair of contributions to the dataset and training, respectively, aimed at better handling the prevalent data disparity, an abundance of unconditioned imaging result features with only a small amount of paired follow-up images. The contributions are as follows.

(1) Self-conditioned learning makes use of the wealth of unpaired images as compared to the small number of paired, longitudinal MRI images by adding the unpaired imaging features to the training dataset and conditioning them on their possibly noised selves.
(2) Gradient manipulation adds robustness in the face of a limited amount of data as well as the handling of two separate tasks through the introduction of self-conditioned learning by manipulating the loss gradient to clip or altogether avoid certain training steps, preventing overfitting and ensuring a smooth training process.

We outline the overall framework and two contributions in more detail below.

### Foundational diffusion framework

Adopting principles from geometric Brownian motion complemented by a stochastic volatility component, we utilize the diffusion process to transform a sample from a simple initial distribution to a more complex target distribution via a series of small diffusive steps.

#### Iterative diffusion

Here, we iteratively add noise to an initial set of imaging features until it resembles a sample from a Gaussian distribution. Then, the features are synthesized by reversing the process and denoising the Gaussian sample step by step until the target output is generated. Formally, our noise addition process for the imaging features at diffusion step *t* is represented by the following stochastic differential equation:

(Equation 1) $$di^{\left(t\right)}=\sqrt{{\hat{\alpha }}_{t}}i^{\left(t\right)}dt+\sqrt{1-{\hat{\alpha }}_{t}}dW^{\left(t\right)}\text{,}$$

where $i^{\left(t\right)}$ denotes the features at time *t*, $\sqrt{\alpha_{\hat{t}}}$ serves as the drift term that scales the feature intensity, $\sqrt{1-\alpha_{\hat{t}}}$ is the diffusion term responsible for scaling the noise, and $dW^{\left(t\right)}$ is an infinitesimal Wiener process representing the random noise *ε*. In our experiments, we perform this diffusion discretized over 100 noise steps, and we set ${\hat{\alpha }}_{t}$ by defining *β* as a 100-step linear spacing between $\beta_{\text{start}}=0.0015$ and $\beta_{\text{end}}=0.02$ with α=1−β and ${\hat{\alpha }}_{t}$ the cumulative product of the first *t* values of *α*.

#### Denoising with the DDPM

The reverse process, from Gaussian to target imaging features, is guided by a neural network called the denoising diffusion probabilistic model (DDPM). The goal is to train this DDPM to predict the reverse denoising process given the noisy features at each time step. We do this by predicting the error between the provided, noised features and the true target features, given that noised imaging result, the time step and any other conditioning information. For our task of generating follow-up MRI imaging features, the target is $i_{2}$, and the conditioning information is the original MRI scan result features, $i_{1}$, and the auxiliary patient labels, l, such as demographic or disease data.

#### UNet autoencoder architecture

We implement our DDPM as a UNet autoencoder, first proposed by Ronneberger et al.^66^ for the task of image segmentation but since becoming the standard architecture for diffusion models. The network consists of both downsampling (encoding) and upsampling (decoding) pathways with skip connections between the two to retain the spatial information at various resolutions. This has been shown to be effective for diffusion models in a number of applications, and its multi-resolution approach is especially valuable in medical imaging tasks, where both global structures and local features need to be captured. We adapt it here for our data shape of one-dimensional imaging feature vectors rather than two- or three-dimensional images by replacing any convolutional layers with linear and attention layers. The overall structure is as follows.

(1) Down-sampling pathway. The initial pathway captures the abstract and salient features of the imaging results, progressively reducing the dimensionality from the initial image representation. The pathway is composed of a series of blocks that each begin with layer normalization before mapping both the current input embedding and the conditioning information down to the desired dimensionality, combining them before using a final linear layer to enrich the combined representation. We use three downsampling blocks with output sizes of 512, 256, and 128.
(2) Residual blocks. Next, a series of residual blocks further refines the downsampled embedding. We use two residual blocks, each consisting of a pair of linear layers with an input and output dimensionality of 128 and followed by a sigmoid linear unit activation function, whose final outputs are added back to the block input.
(3) Upsampling pathway (decoding). The imaging feature vector is then reconstructed to its initial dimensions. This pathway consists of blocks that largely mirror those in the downsampling pathway but that further combine the encoding pathway’s intermediate features via skip connections along with the current representation and conditioning info. We use three upsampling blocks with output sizes of 256, 512, and the original data dimensionality to match the input sizes of each of the corresponding downsampling blocks.
(4) Time-step embedding and conditioning. To inform and guide the autoencoding process, both temporal and auxiliary conditioning information are embedded and passed into the encoding and decoding blocks. First, the current time step in the diffusion process is positionally encoded using sinusoidal functions, normalized by the total number of diffusion steps. In contexts where additional conditioning data are present, such as in our primary task with $i_{1}$ and l, these data undergo an additional embedding process to match the positional embedding dimensionality. The temporal and auxiliary embeddings are then summed, and this composite embedding is used to steer the autoencoding throughout the model.

#### Model usage

The UNet architecture is trained to predict the error between a noise-infused imaging feature vector and the corresponding true target vector. This prediction is informed by both the time step associated with the noising process and any relevant conditioning data. Upon successful training, the generation process commences with an input initialized as pure Gaussian noise. By iteratively feeding this input, along with the patient’s initial MRI and the associated auxiliary labels to the trained model, we repeatedly refine the output and generate realistic follow-up MRI results.

### Self-conditioned framework

We then add our two proposed augmentations to that standard conditional diffusion framework. Given the scarcity of paired, follow-up MRI images and a larger set of unpaired images, the traditional training approach of only using the longitudinal data that matches our task may result in a severe under-utilization of the available data. We propose the introduction of self-conditioned learning to solve this problem. Specifically, we include the unpaired imaging features for training our diffusion model by conditioning them on a possibly perturbed version of themselves in addition to the auxiliary patient labels.

In our self-conditioned framework, we utilize a combination of true longitudinal conditioning and the proposed self-conditioning approach. When a true follow-up imaging feature vector, $i_{2}$, is available for a particular sample, the target is set to that and conditioned on the genuine conditioning information $i_{1}$ and l so that the model is tasked with our standard learning task of approximating $P\left(i_{2}|i_{1},l\right)$. However, in the absence of that follow-up imaging feature vector, we instead set the target vector to be $i_{1}$ and condition the model on l along with either the same $i_{1}$ value or a slightly perturbed version, $\tilde{i_{1}}$, with a small amount of Gaussian noise equivalent to 5 diffusion steps (which is a hyperparameter determined experimentally) added. This stochastic choice between $i_{1}$ and $\tilde{i_{1}}$ in this self-conditioning case serves to add further diversity to the learning inputs as a form of data augmentation. More formally, this self-conditioning transforms the task to learning $P\left(i_{t}|i_{s},l\right)$, where $i_{t}$ is

(Equation 2) $$i_{t}=\left\{ \begin{array}{cc} i_{2} & \text{if}\;i_{2}\;\text{is}\;\text{available}\\ i_{1} & \text{otherwise} \end{array}\right.$$

and $i_{s}$ is

(Equation 3) $$i_{s}=\left\{ \begin{array}{cc} i_{1} & \text{if}\;i_{2}\;\text{is}\;\text{available}\\ i_{1} & \text{if}\;i_{2}\;\text{is}\;\text{not}\;\text{available}\;\text{and}\;\delta =0\\ \tilde{i_{1}} & \text{if}\;i_{2}\;\text{is}\;\text{not}\;\text{available}\;\text{and}\;\delta =1 \end{array}\right.$$

where δ∼Bernoulli(0.5) is a Bernoulli random variable with equal probability of being 0 or 1. These true and self-conditioned samples are then combined into one large, diverse training dataset.

This approach is designed to offer a pair of key benefits. The first is more simple data enrichment. Instead of relying on just the small amount of real pairs and learning from the small corresponding training set, the introduction of self-conditioned data allows the model to be exposed to an extended set of scenarios, making it more robust and versatile while preventing overfitting too early. The second advantage is the slightly more nuanced concept of deferred learning. The additional robustness offered by more data is especially important given that the task involves two different types of learning. The model needs to both learn the patterns of overall MRI imaging feature generation (learning P(i)) and the patterns of longitudinal MRI progression (our main task of learning $P\left(i_{2}|i_{1},l\right)$). The self-conditioned samples allow our model to better learn the first set of patterns so that it can subsequently better learn the second set of patterns before overfitting, and they allow it to do so all at once without any task shift, as would be produced in the case of pretraining.

### Training gradient manipulation

In our second proposed contribution, we utilize direct manipulation of the gradient from our loss function comparing the true and predicted errors, as discussed in the diffusion framework. This allows us to combat overfitting and smooth the learning process more evenly over the many iterative training steps. This is especially valuable given the limited and heterogeneous data introduced by our problem formulation and the self-conditioned training. Such challenges can lead to convergence issues and model instability, where gradients of large magnitude lead to catastrophic updates, which we aim to prevent.

#### L2 gradient norm calculation

The first step is to calculate the L2 gradient norm over the model’s parameters after each forward and backward pass. Given our loss function *L* and model parameters *θ*, the L2 norm, denoted as *G*, is computed by

(Equation 4) $$G=\sqrt{\sum_{i=1}^{N}{\left(\frac{\partial L}{\partial \theta_{i}}\right)}^{2},}$$

where *N* represents the total number of parameters in the model. This then signifies the magnitude of the intended gradient update (while the direction is left to the composition of individual derivatives).

#### Gradient thresholding

Given the calculation of such norms for one preliminary pass through the dataset, we then derive a pair of threshold values, $\delta_{\max}$ and $\delta_{err}$, based on the average and maximum gradient magnitude after one pass through the dataset. Specifically, we set the maximum step size, $\delta_{\max}$, to the mean L2 gradient norm, and we set the threshold at which a gradient is decided to be erroneous (or at least unstable), $\delta_{err}$, to three times the largest norm from our initial pass. We found this multiplier value of 3 to be effective, but it is a hyperparameter that can be fine-tuned.

#### Gradient manipulation

Finally, we use these thresholds to manipulate the gradients during training, which are used to update our model. Specifically, given a gradient component, $g_{i}$, for parameter $\theta_{i}$ and the current overall L2 gradient norm *G*, we calculate the final adjusted gradient component by

(Equation 5) $$g_{i}^{\prime }=\left\{ \begin{array}{cc} g_{i} & \text{if}\;G\leq \delta_{\max}\\ g_{i}\times \frac{\delta_{\max}}{G} & \text{if}\;\delta_{\max}<G\leq \delta_{err}\\ 0 & \text{otherwise} \end{array}\right.\text{.}$$

So, we skip gradients that are above our $\delta_{err}$ threshold, and we ensure that all other gradients are no greater than $\delta_{\max}$ through gradient norm clipping. We experimentally find and use gradient thresholds of $\delta_{\max}=0.3$ and $\delta_{err}=0.75$.

In practice, only a small number of gradients are skipped, but we found experimentally that allowing these updates through, even in a clipped format, led to the training derailing and the model unlearning much of what the training up to that point had achieved. Thus, skipping them altogether ensures robustness against such potential catastrophic steps at minimal cost, particularly valuable when learning from data of disparate quality, as may be introduced by our self-conditioned framework. The general gradient norm clipping then aims to ensure a stable and efficient training process, combatting overfitting and achieving better generalization by normalizing the size of different steps. By introducing this gradient manipulation into the training process, our model becomes more resilient to potential instabilities, optimizing the learning trajectory in the high-dimensional space and allowing our model to make the most of every gradient update, mitigating the negative effects of potentially harmful updates while benefitting from the informative ones.

**Figure 3:**
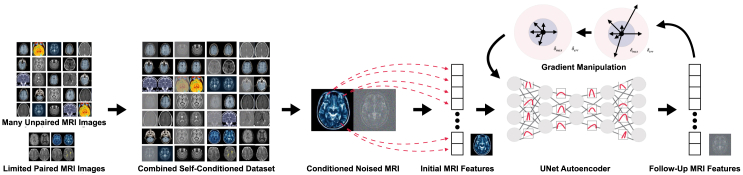
SECONDGRAM architecture The SECONDGRAM framework is visualized. SECONDGRAM uses a neural diffusion setup with a combined, partially self-conditioned dataset, a UNet Autoencoder, and a gradient-manipulated training regime to add robustness and to increase the amount of training data. This allows the model to better learn the patterns of MRI progression in the face of a scarcity of paired, longitudinal imaging results.

In conclusion, the proposed SECONDGRAM combines the power of diffusion models with gradient-manipulated robustness and self-conditioned learning to address the challenge of generating missing follow-up MRI imaging features. The diffusion model provides the foundational architecture; the gradient manipulation ensures smooth, stable training; and self-conditioning boosts learning efficacy, culminating in the comprehensive end-to-end process shown in Figure 3.

## Resource availability

### Lead contact

Requests for further information and resources should be directed to and will be fulfilled by the lead contact, Jimeng Sun (jimeng@illinois.edu).

### Materials availability

This study did not generate new materials.

### Data and code availability

• The UK Biobank dataset^26^ that we use is hosted by DNAnexus on a research access platform that is publicly available to researchers and may be downloaded and used freely after an application process at https://ams.ukbiobank.ac.uk/ams/.
• All original code has been deposited at Zenodo (https://doi.org/10.5281/zenodo.14726354) and is publicly available there as well as at https://github.com/btheodorou99/SECONDGRAM/as of the date of publication.^67^
• Any additional information required to reanalyze the data reported in this paper is available from the lead contact upon request.

## Acknowledgments

This work was supported by NSF awards SCH-2205289, SCH-2014438, and IIS-2034479. This research was also supported in part by the Intramural Research Program of the NIH, 10.13039/100000049National Institute on Aging, National Institutes of Health, Department of Health and Human Services, project number ZO1 AG000534, as well as the 10.13039/100000065National Institute of Neurological Disorders and Stroke.

## Author contributions

B.T. and A.D. proposed the method, B.T. conducted all the experiments, and B.T., A.D., M.N., J.S., and F.F. wrote the manuscript.

## Declaration of interests

Some authors’ participation in this project was part of a competitive contract awarded to DataTecnica LLC by the National Institutes of Health to support open science research. M.N. also owns stock in Character Bio Inc. and Neuron23 Inc.
